# The Diagnostic and Prognostic Utility of Heat Shock Protein 27 as a Biomarker for Oral Squamous Cell Carcinoma

**DOI:** 10.7759/cureus.113413

**Published:** 2026-07-26

**Authors:** Katy Flannery, Maryam Salik, Hilal Arnouk

**Affiliations:** 1 Pathology, Midwestern University Chicago College of Osteopathic Medicine, Downers Grove, USA; 2 Pathology and Laboratory Medicine, Midwestern University Chicago College of Osteopathic Medicine, Downers Grove, USA; 3 Pathology, Illinois College of Osteopathic Medicine, The Chicago School, Chicago, USA

**Keywords:** diagnostic biomarker, head and neck cancer, hsp27, oral squamous cell carcinoma, prognostic biomarker, tumor progression

## Abstract

Objective

Despite multiple studies on the involvement of heat shock protein 27 (HSP27) in cancer progression in oral squamous cell carcinoma (OSCC), there is a lack of overall consensus on its expression trends in this type of cancer. In this study, we examined the differential expression of HSP27 in tissue samples of oral cancer and the normal oral mucosa. Additionally, we correlated HSP27 expression with crucial clinicopathological parameters, including clinical staging and histopathological grading. Our findings suggest that HSP27 is a potential biomarker that can aid in the diagnosis and prognosis of patients suffering from oral cancer.

Study design

Immunohistochemistry was performed to detect HSP27 in both normal oral mucosa and OSCC from a variety of clinical stages and histopathological grades. Samples then underwent imaging followed by computer-assisted image analysis to objectively quantify the data.

Results

The HSP27 immunohistochemical detection coupled with computer-assisted image analysis documented a significant increase in HSP27 expression in OSCC tissue samples compared to normal oral mucosa tissue samples. Its expression analysis also showed correlations with the nodal status, clinical stage, and histopathological grade.

Conclusion

This study examined HSP27 expression patterns in oral cancer tissue samples and found HSP27 to be upregulated in OSCC compared to the normal oral mucosa. Additionally, HSP27 expression significantly correlated with the histopathological grade, nodal status, and the overall clinical stage. This study paves the way for the potential use of HSP27 as a supplementary diagnostic and prognostic tool in evaluating and monitoring disease progression in oral cancer patients.

## Introduction

Oral squamous cell carcinoma (OSCC) constitutes the majority of head and neck cancer cases [1]. Overall, cases of cancer affecting the lip and oral cavity have the 16th highest incidence, with about 390,000 cases, and rank 15th for cancer mortality with about 189,000 deaths worldwide [2]. Over the past two decades, there has been a steady increase in the incidence of oral cavity cancers [2]. Oral squamous cell carcinoma develops in the oral cavity and oropharynx and can be linked to several different etiological factors, including cigarette smoking and alcohol [3]. Despite its prevalence, OSCC has seen little improvement in prognosis, with modest changes in mortality rates over the past few decades [2,4].

Early detection and accurate diagnosis of OSCC are challenging due to its morphological resemblance to benign and inflammatory lesions in the oral cavity [5]. The current standard for OSCC diagnosis relies on biopsy and histopathological analysis [6], which relies on examining the tissue morphology alone, allowing for variability amongst examiners. Several studies have aimed to identify biomarkers for OSCC and found correlations with ubiquitous cancer biomarkers, including the tumor suppressors PTEN and p53, as well as with more unique biomarkers, such as DJ-1 and Cornulin [7-9], although their translation into clinical applications has been lagging in most cases. As the incidence of oral cancer continues to rise [2] and delayed diagnosis remains a hurdle to achieving better clinical outcomes, it is vital that new approaches to determining diagnosis and prognosis, including the use of molecular biomarkers, continue to be explored.

Heat shock protein 27 (HSP27) is a member of the heat shock protein family and functions as a molecular chaperone that has a cellular protective role, preventing cell damage from heat shock and oxidative stress [10]. It also plays an anti-apoptotic role in the Fas pathway, inhibiting Daxx-dependent apoptosis [11]. Given its various regulatory roles, including the response to cellular stress, HSP27 is poised to have an important role in tumorigenesis and the progression of human cancers [12,13]. Specifically, HSP27 is involved in the pathogenesis of different cancers stemming from the squamous epithelium [14]. Despite multiple studies on the involvement of HSP27 in cancer progression in OSCC, there is a lack of overall consensus on its expression trend in this type of cancer. Several studies show upregulation of HSP27 in OSCC when compared to the normal oral mucosa [15,16], while other studies found reduced expression of HSP27 in OSCC compared to normal oral mucosa [17] along with a documented poor survival outcome associated with the reduced HSP27 expression [18,19].

In this study, we examined the differential expression of HSP27 in tissue samples of oral cancer and the normal oral mucosa. Additionally, we correlated HSP27 expression with crucial clinicopathological parameters, including clinical staging and histopathological grading, using an immunohistochemical detection assay paired with objective computer-assisted quantitative analysis. Our findings suggest that HSP27 is a potential molecular biomarker that can aid in the diagnosis and prognosis of patients suffering from oral cancer.

## Materials and methods

Tissue samples

The tissue samples for this study consist of de-identified tissue microarray samples provided by TissueArray.com (Derwood, MD, USA) and Pantomics (Fairfield, CA, USA). A total of 193 tissue samples were utilized for our analysis, including 22 samples representing the normal oral mucosa and 171 representing OSCC with varying histopathological grades and TNM parameters. During the detection assay and initial analysis, the experimenters were blinded to all characteristics of the tissue samples, including whether the samples were normal oral mucosa or OSCC and their histopathological grade and the tumor, node, and metastasis (TNM) classification. Tissue microarrays underwent deparaffinization in preparation for immunohistochemistry.

Immunohistochemistry staining

The tissue samples underwent antigen retrieval with whole citrate buffer, followed by blocking with bovine serum albumin (BSA). The tissue samples were incubated with a 1:500 diluted HSP27 rabbit polyclonal antibody (Proteintech, Rosemont, IL, USA) at 23 degrees Celsius for 90 minutes, followed by incubation with a 1:2000 diluted anti-rabbit IgG peroxidase-conjugated secondary antibody (Sigma-Aldrich, St. Louis, MO, USA) at 23 degrees Celsius for 60 minutes. Samples were then incubated with 3,3’ diaminobenzidine (DAB) chromogenic substrate at 23 degrees Celsius for five minutes and then counterstained with hematoxylin. Once dried, samples were mounted with a coverslip for preservation.

Quantitative analysis

The immunohistochemistry-stained tissue samples were imaged with a high-resolution Nikon A1R (Nikon Corp., Tokyo, JPN) inverted microscope at the 10x magnification setting. The microscope underwent adjustments for standardization and optimization prior to formal imaging, and images were taken in one session to ensure consistency and avoid introducing any technical variation. Following imaging, Aperio ImageScope software (Leica Biosystems Inc., Buffalo Grove, IL, USA) was utilized for computer-assisted image analysis to generate objective and consistent quantitative data and to eliminate the subjectivity and bias typically associated with manual scoring.

Quantification of immunoreactivity was done with a positive pixel counting v9 algorithm from the Aperio ImageScope software. First, samples underwent manual selection of regions of interest to ensure only tumor parenchyma and normal oral mucosa were being analyzed, excluding analysis of the stroma and adjacent connective tissues. Strong, medium, and weak positive pixels were assigned red, orange, and yellow colors, respectively, and negative pixels were assigned the color blue by the Aperio ImageScope software. The immunoreactivity of each sample was quantitatively determined as a histo-score (H-score) based on the percentage of positively stained epithelial cells and the intensity of that staining. The formula used was as follows: H-score = (1 × % weak positivity) + (2 × % medium positivity) + (3 × % strong positivity).

Statistical analysis

Tissue samples were sorted into different groups based on the clinicopathological parameters in each comparison before their H-scores were averaged for the group. Once sorted, the mean, standard deviation, and standard error of the mean (SEM) were calculated for each comparison group to quantify the precision of the mean in each group. Then an unpaired t-test was applied to each of the two compared groups using the GraphPad software (GraphPad Software Inc., San Diego, CA, USA) to determine the statistical significance. The p-value was generated, and p < 0.05 was set as the threshold for a statistically significant correlation.

## Results

Differentially expressed HSP27 in OSCC and normal oral mucosa tissues

**Figure 1:**
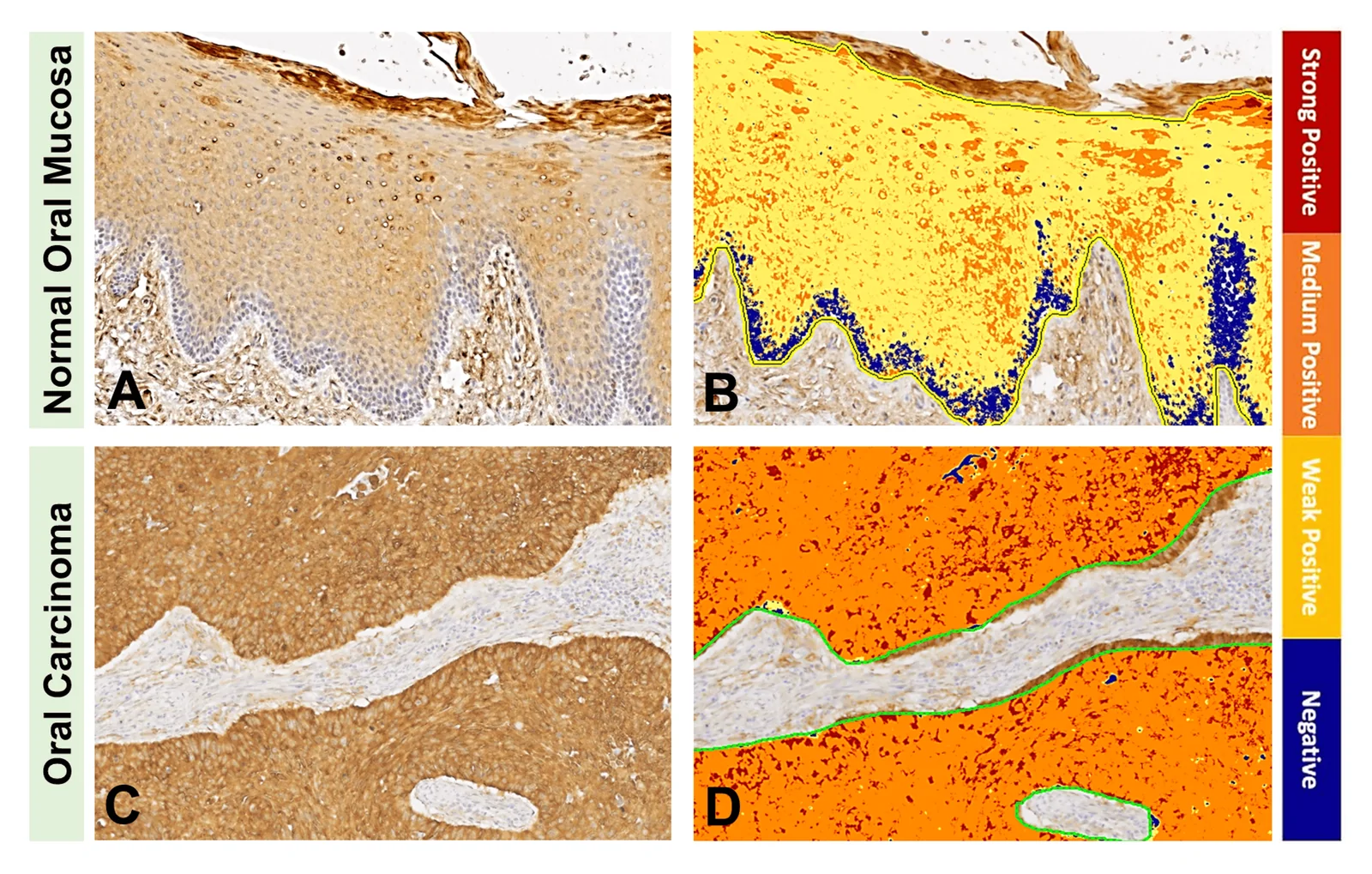
HSP27 expression in normal oral mucosa samples vs. OSCC samples (immunohistochemistry (IHC) 200×) The upper panel shows representative images of a normal oral mucosa tissue sample: (A) IHC with brown staining indicating immunoreactivity of HSP27, (B) ImageScope computer-assisted analysis (Leica Biosystems Inc., Buffalo Grove, IL, USA). The lower panel shows representative images of an OSCC tissue sample: (C) IHC with brown staining indicating immunoreactivity of HSP27, and (D) ImageScope computer-assisted analysis. HSP27: Heat shock protein 27, OSCC: Oral squamous cell carcinoma, IHC: Immunohistochemistry

The HSP27 immunohistochemical detection coupled with computer-assisted image analysis documented a notable increase in HSP27 expression in OSCC tissue samples compared to normal oral mucosa tissue samples (Figure 1).

**Figure 2:**
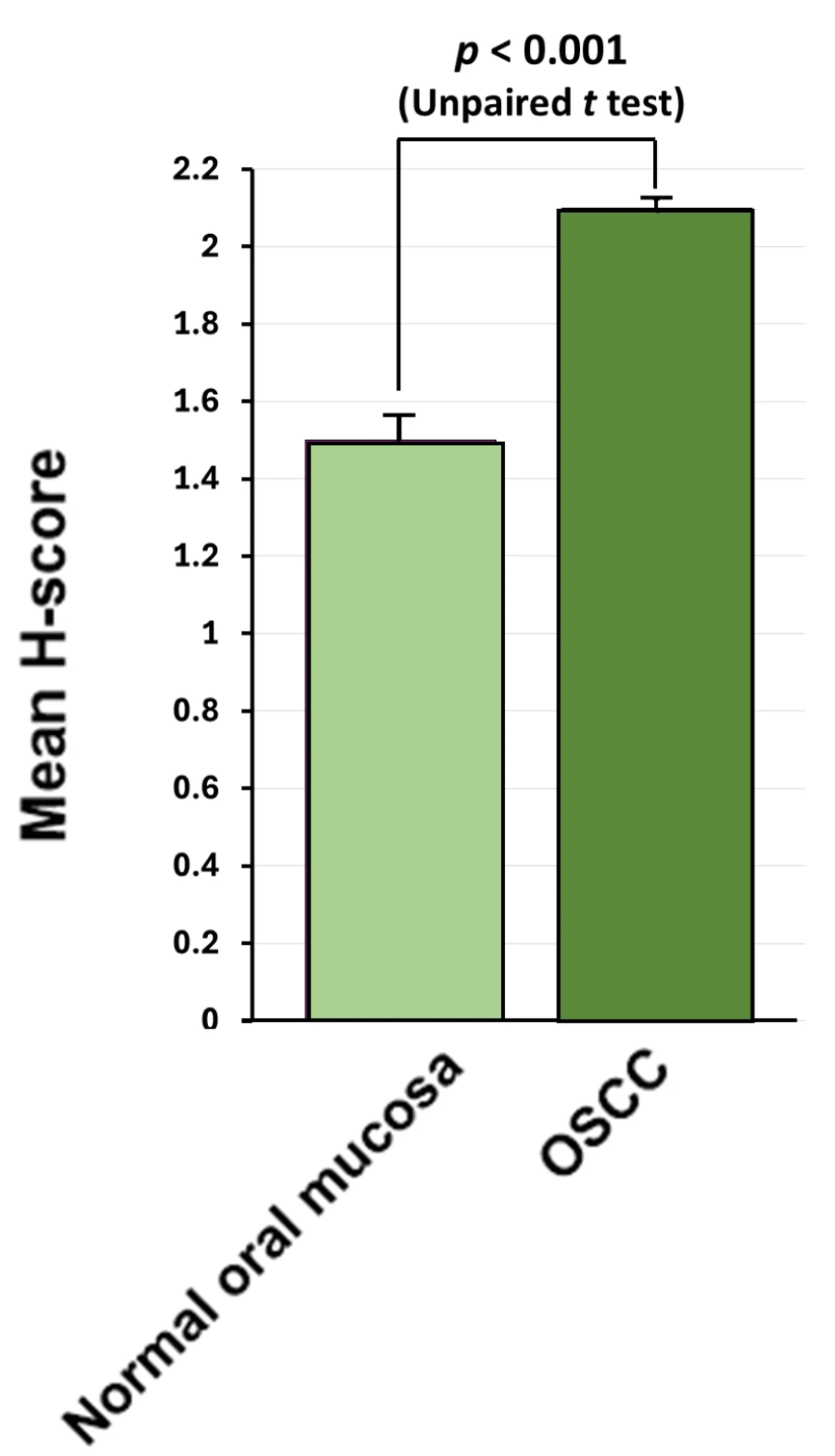
Differential HSP27 expression in normal oral mucosa samples vs. OSCC samples Bar graph depicting the mean H-score of HSP27 expression in normal oral mucosa (n=22) and OSCC (n=171). Error bars represent the SEM; the unpaired t-test was used for comparison, and a p-value* *<0.05 is considered significant. HSP27: Heat shock protein 27, OSCC: Oral squamous cell carcinoma, SEM: Standard error of the mean

Quantitatively, the normal oral mucosa samples (n=22) showed an average H-score of 1.5, while the average H-score was 2 in OSCC samples (n=171). This upregulation in HSP27 expression was statistically significant (p < 0.001) (Figure 2).

Correlation of HSP27 expression with lymph node involvement status

**Figure 3:**
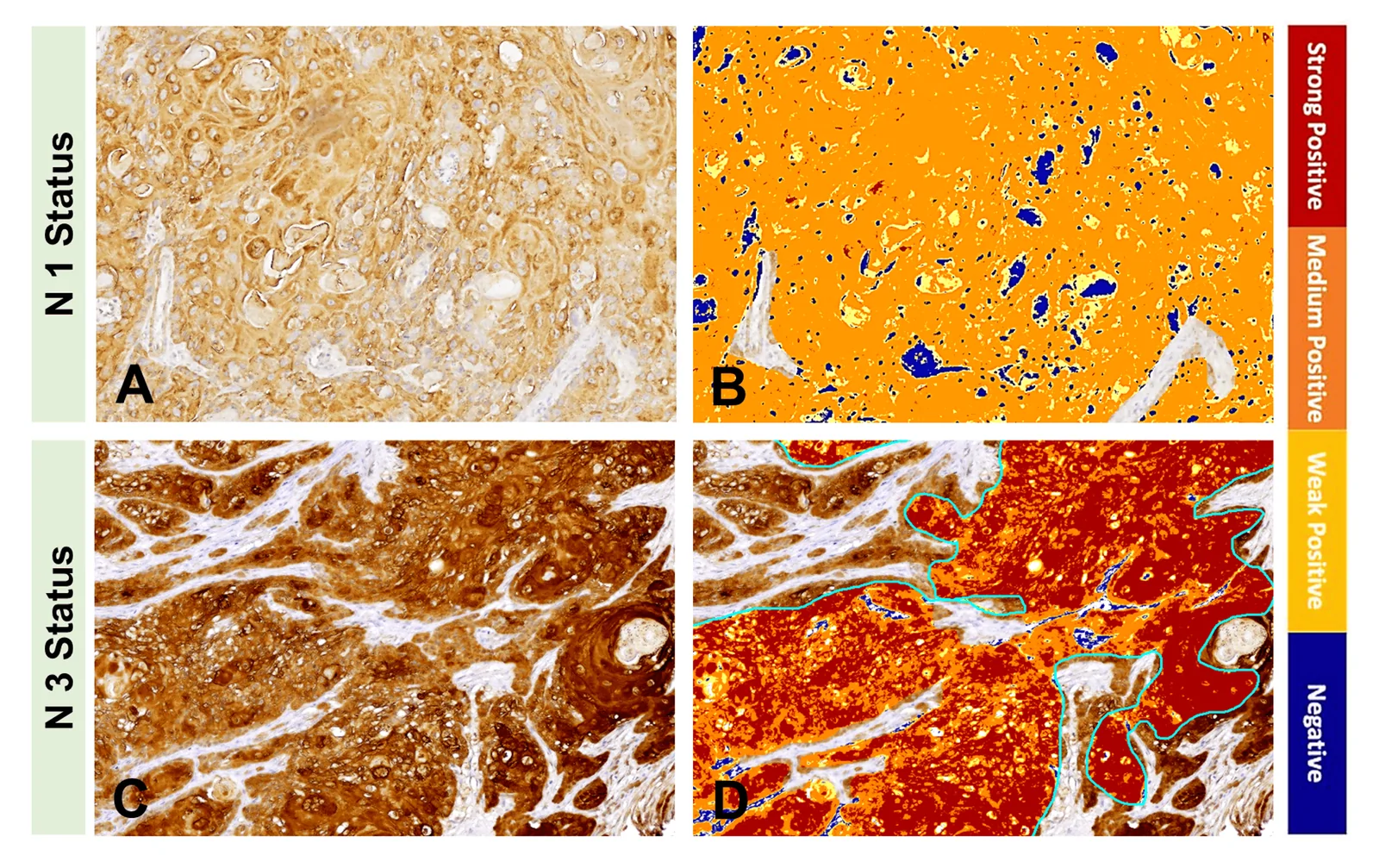
HSP27 expression in tissue samples of OSCC with variable nodal status (IHC, 200×) The upper panels show representative images of an OSCC tissue sample with nodal status N1: (A) IHC with brown staining indicating immunoreactivity of HSP27, (B) ImageScope computer-assisted analysis (Leica Biosystems Inc., Buffalo Grove, IL, USA). The lower panels show representative images of an OSCC tissue sample with nodal status N3: (C) IHC with brown staining indicating immunoreactivity of HSP27, and (D) ImageScope computer-assisted analysis HSP27: Heat shock protein 27, OSCC: Oral squamous cell carcinoma, IHC: Immunohistochemistry

The analysis of HSP27 expression revealed a correlation with the nodal status. Overall, tissue samples with limited to no lymph node involvement (N0 and N1) showed more HSP27 immunoreactivity than samples from patients with increased lymph node involvement (Figure 3).

**Figure 4:**
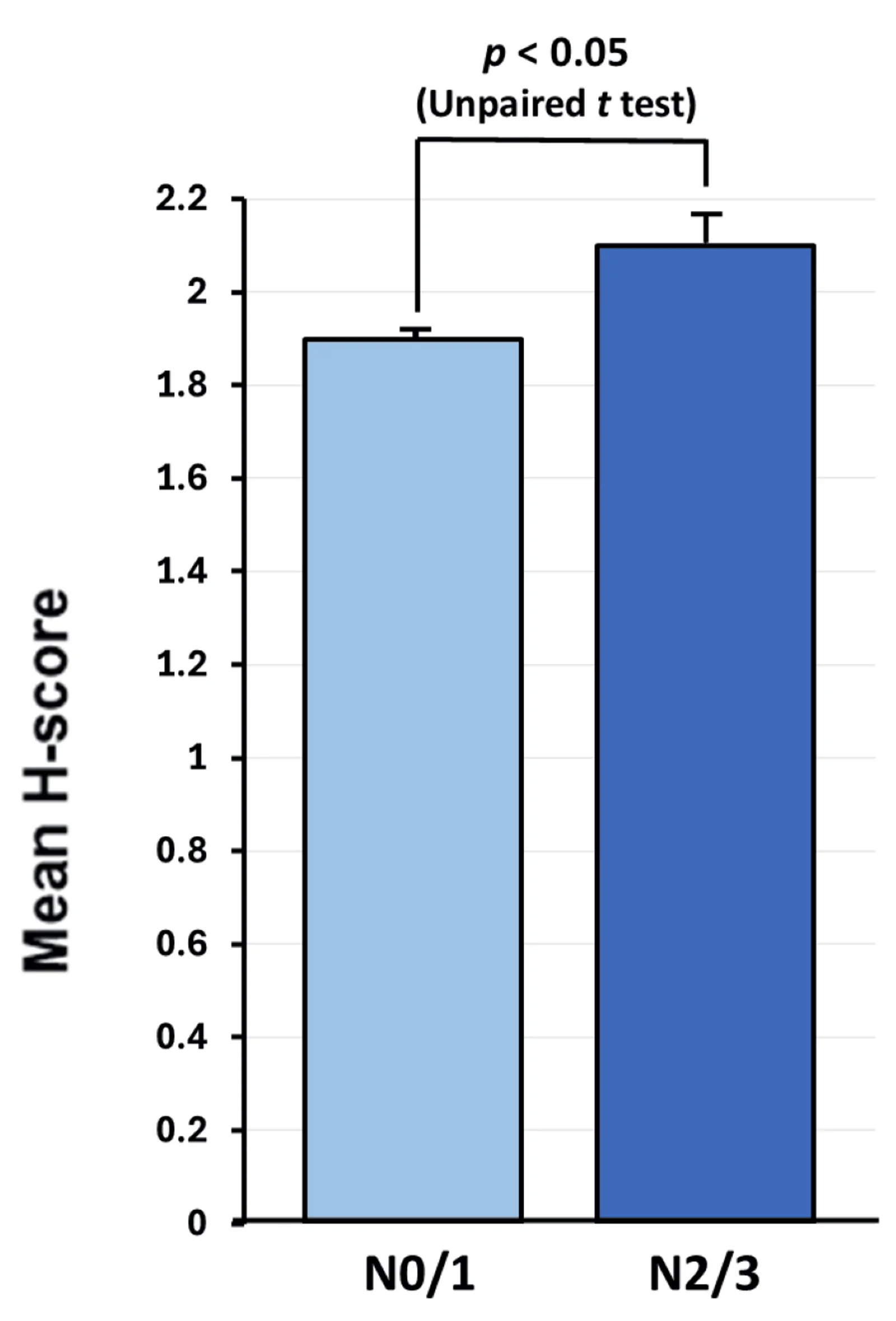
Correlation between HSP27 expression and the nodal status in tissue samples of OSCC Bar graph depicting the mean H-score of HSP27 expression in N0/N1 OSCC samples (n=147) and N2/N3 OSCC samples (n=22). Error bars represent SEM; the unpaired t-test was used for the comparison, and a p-value <0.05 is considered significant. HSP27: Heat shock protein 27, OSCC: Oral squamous cell carcinoma, SEM: Standard error of the mean, NO/N1: Limited to no lymph node involvement, N2/N3: Increased lymph node involvement

Quantitatively, tissue samples with limited to no lymph node involvement (N0 and N1) had an average H-score of 1.9 (n=147), while samples with increased lymph node involvement (N2 and N3) had an average H-score of 2.1 (n=22). This correlation was statistically significant (p < 0.05) (Figure 4).

Correlation of HSP27 expression with TNM clinical staging

**Figure 5:**
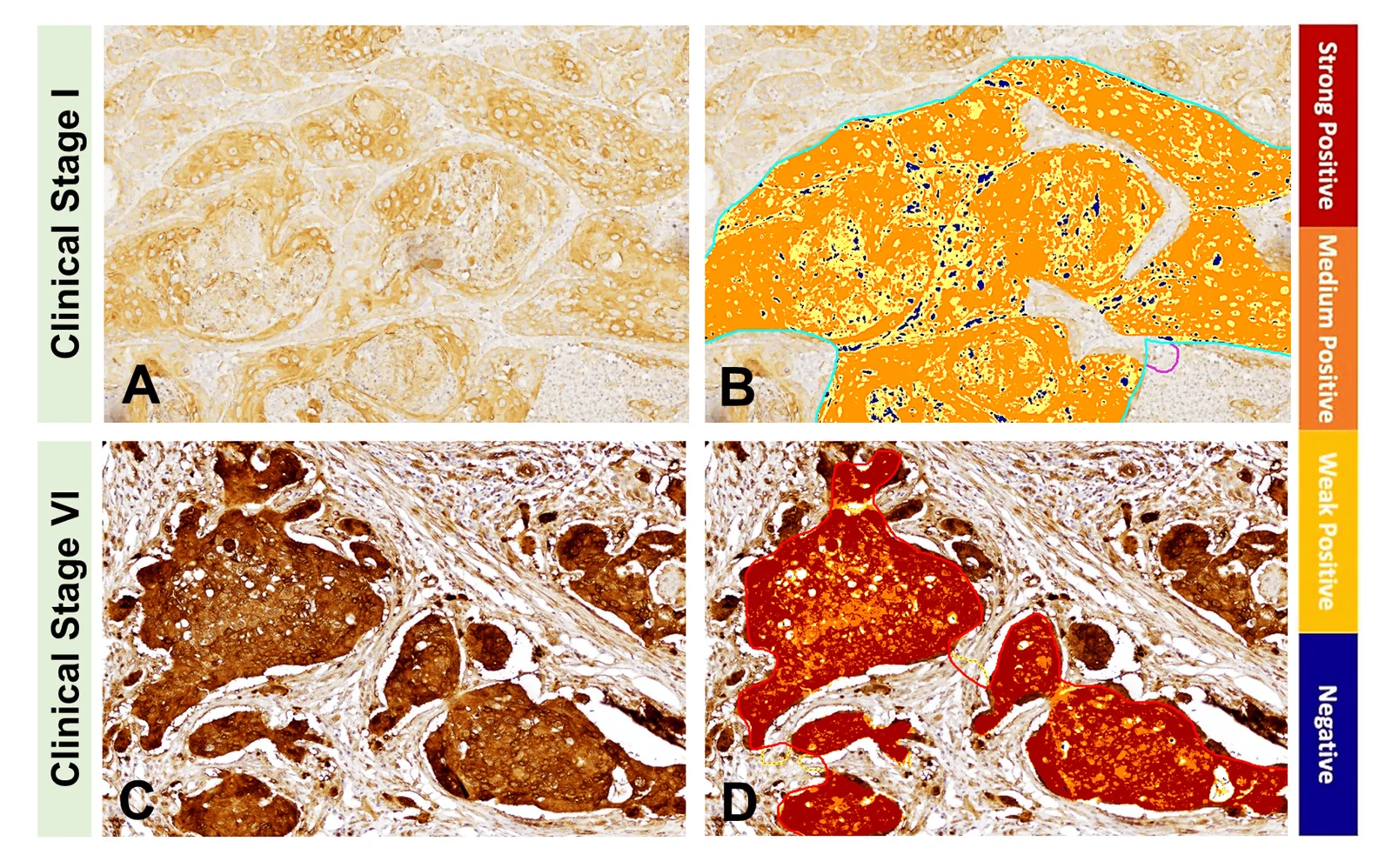
HSP27 expression in early clinical stages (I/II) vs. late clinical stages (III/IV) of OSCC (IHC, 200×) The upper panels show representative images of an early-stage I OSCC tissue sample: (A) IHC with brown staining depicting immunoreactivity of HSP27, (B) ImageScope computer-assisted analysis (Leica Biosystems Inc., Buffalo Grove, IL, USA). The lower panels show representative images of a stage IV OSCC tissue sample: (C) IHC with brown staining depicting immunoreactivity of HSP27, and (D) ImageScope computer-assisted analysis. HSP27: Heat shock protein 27, OSCC: Oral squamous cell carcinoma, IHC: Immunohistochemistry

Image analysis of HSP27 immunoreactivity revealed a correlation with TNM clinical staging. Overall, tissue samples from OSCC patients with early stages I and II showed lower levels of HSP27 expression than tissue samples from OSCC patients with late stages III and IV of OSCC (Figure 5).

**Figure 6:**
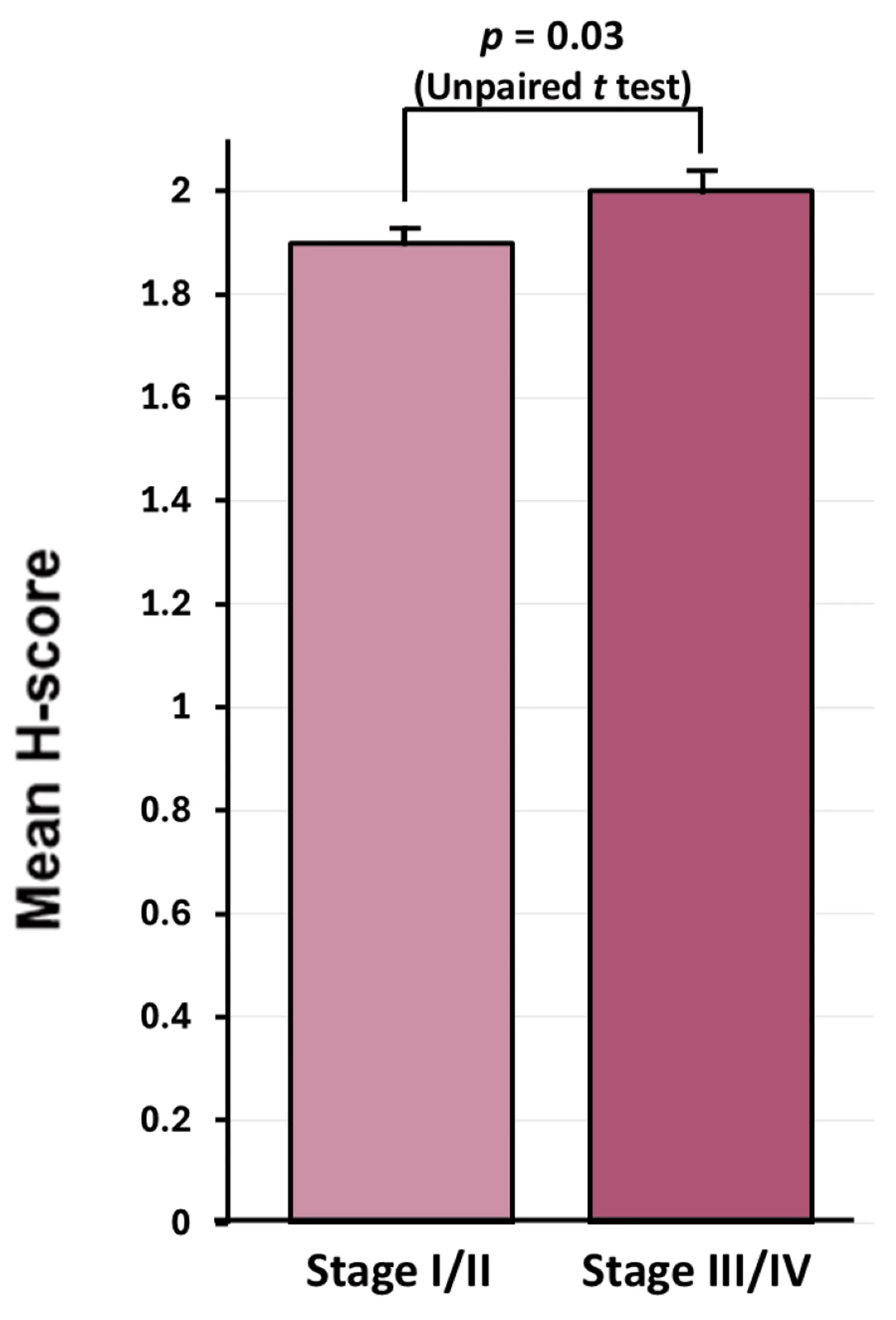
Correlation between HSP27 expression and clinical staging in tissue samples of OSCC Bar graph showing the mean H-score of HSP27 expression in the early clinical stages I/II OSCC samples (n=85) and the late clinical stages III/IV OSCC samples (n=84). Error bars represent SEM; the unpaired t-test was used for the comparison, and a p-value <0.05 is considered significant. HSP27: Heat shock protein 27, OSCC: Oral squamous cell carcinoma, SEM: Standard error of the mean

Computer-assisted analysis documented an average H-score of 1.9 for OSCC tissue samples from patients with the early stages I and II (n=85) and an average H-score of 2 for OSCC samples from patients with the late stages III and IV of OSCC (n=84). The correlation was statistically significant with a p-value of 0.03 (Figure 6).

Correlation of HSP27 expression with histopathological grading

**Figure 7:**
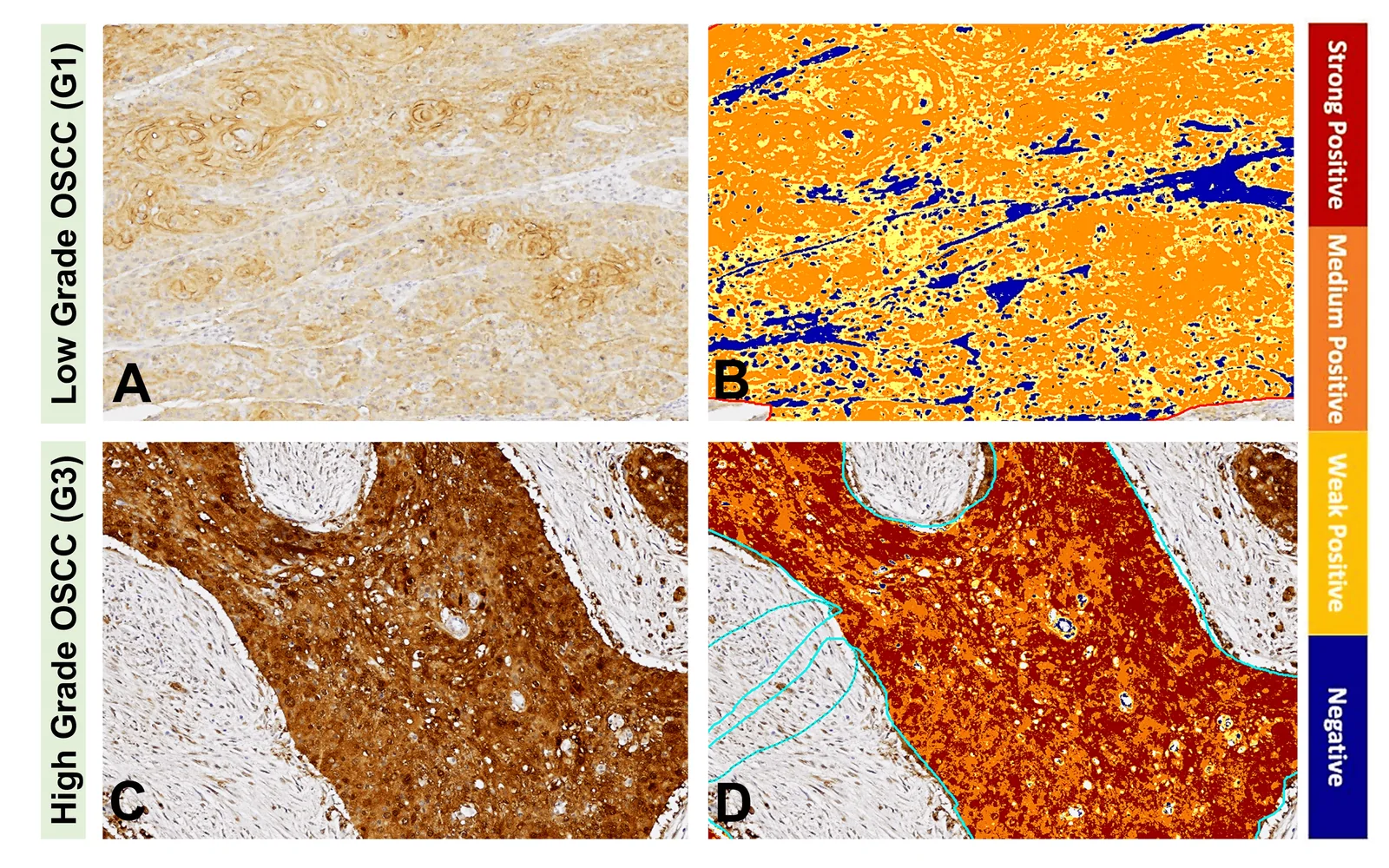
HSP27 expression in tissue samples of OSCC with low histopathological grade vs. high histopathological grades (IHC, 200×) The upper panels show representative images of an OSCC tissue sample with low histopathological grade 1 (G1): (A) IHC with brown staining depicting immunoreactivity of HSP27, (B) ImageScope computer-assisted analysis (Leica Biosystems Inc., Buffalo Grove, IL, USA). The lower panels show representative images of an OSCC with high histopathological grade 3 (G3) OSCC tissue samples: IHC with brown staining depicting immunoreactivity of HSP27, (D) ImageScope computer-assisted analysis. HSP27: Heat shock protein 27, OSCC: Oral squamous cell carcinoma, IHC: Immunohistochemistry

Computer-assisted image analysis of HSP27 immunoreactivity revealed a correlation between HSP27 expression and the histopathological grade. Specifically, OSCC samples characterized by low histopathological grade (grade 1) demonstrated lower HSP27 expression compared to OSCC samples with high histopathological grades 2 and 3 (Figure 7).

**Figure 8:**
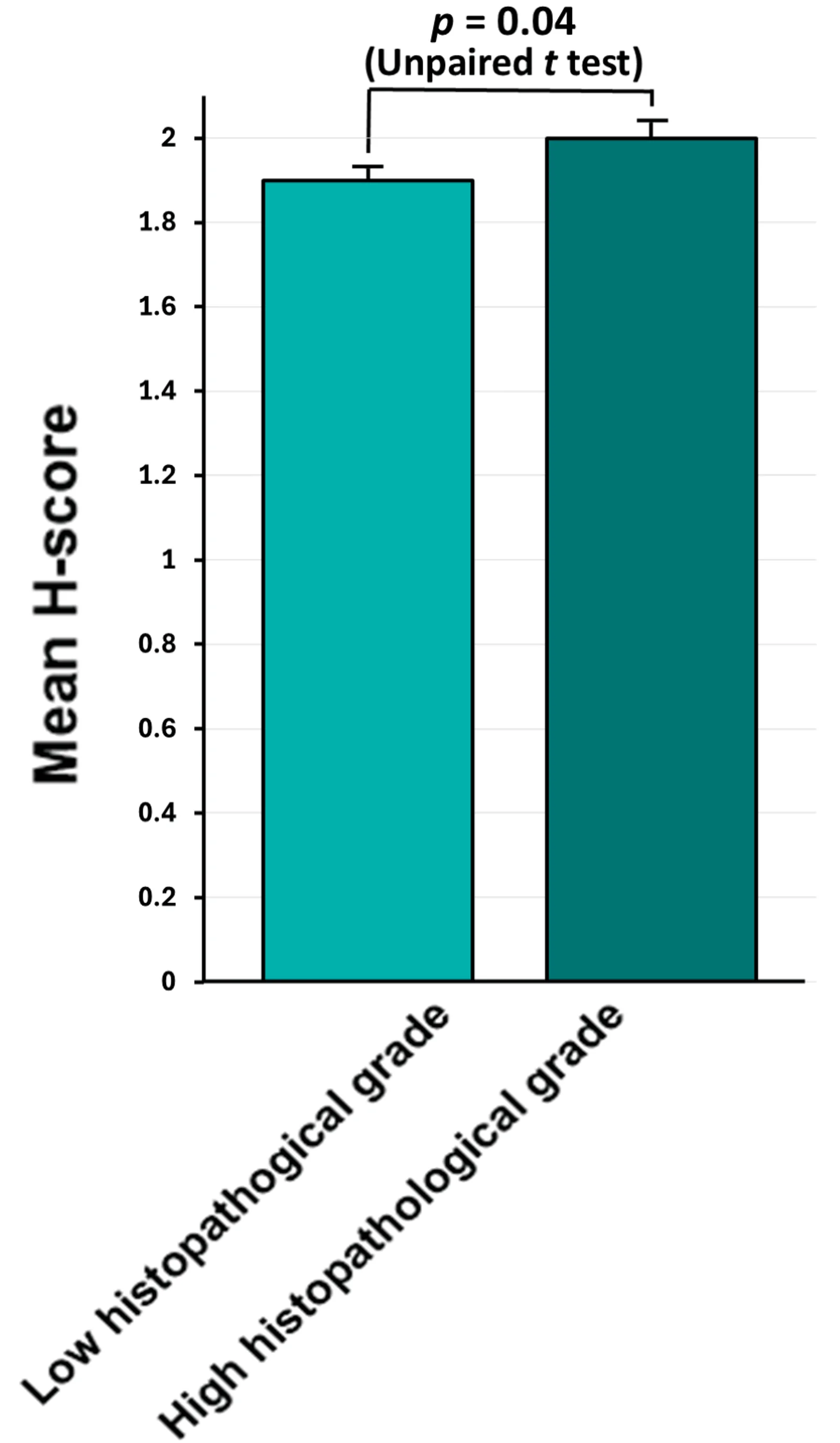
Correlation between HSP27 expression and histopathological grading Bar graph showing the mean H-score of HSP27 expression in the low histopathological grade (G1) OSCC samples (n=88) versus the high histopathological grades (G2/G3) OSCC samples (n=67). Error bars represent SEM; the unpaired t-test was used for the comparison, and a p-value <0.05 is considered significant. HSP27: Heat shock protein 27, OSCC: Oral squamous cell carcinoma, SEM: Standard error of the mean, G1: Grade 1, G2: Grade 2, G3: Grade 3

Quantitative analysis documented that the average H-score was 1.9 for tissue samples with low histopathological grade (n=88), while high histopathological grade tissue samples had an average H-score of 2.0 (n=67). This comparison was statistically significant with a p-value of 0.04 (Figure 8).

## Discussion

As a molecular chaperone, HSP27 is involved in cellular stress responses and other protective activities [10,20]. Heat shock protein 27 is frequently found to be upregulated in malignant neoplasms, including cancers of the squamous epithelium, such as esophageal squamous cell carcinoma and cervical squamous cell carcinoma [14,21-23]. This study evaluated the use of HSP27 as a diagnostic biomarker for OSCC. We found that there is a significant upregulation in HSP27 expression in OSCC tissues compared to normal oral mucosa.

Additionally, this study also evaluated HSP27 prognostic utility in OSCC. We found that there is significantly greater expression of HSP27 in samples with higher histopathological grades compared to samples with lower histopathological grades. This is significant as higher histopathological grades tend to correlate with poor survival outcomes and increased recurrence [24].

Moreover, HSP27 expression correlated with the nodal status. We documented a significant increase in HSP27 expression in tissue samples with a more advanced lymph node involvement (N2 and N3) compared to samples with little to no lymph node involvement (N0 and N1). Additionally, HSP27 expression in tissue samples was significantly greater in the late clinical stages (stage III and stage IV) compared to the early clinical stages (stage I and stage II). This correlation is clinically relevant given the drastic difference in the five-year survival rates between OSCC patients with early clinical stages (over 80%) and OSCC patients with late clinical stages (under 45%) [25]. We have shown that HSP27 expression is correlated with the crucial prognostic indicators of the TNM clinical staging and histopathological grading, which suggests that HSP27 expression can potentially be used as a supplementary biomarker to aid in the detection and prognosis of oral cancers and to monitor tumor progression and response to therapy. 

Limitations

In the present study, we utilized tissue microarrays because they provided the advantage of sampling and processing multiple tissue samples simultaneously, which reduced experimental variability and allowed for a more uniform comparative analysis. However, they somewhat limited the scope of the study since tissue microarrays consist of small cores of tissue samples that may not accurately represent the entirety of heterogeneous tumors. Moreover, tissue microarray samples tend to be archival, leading to a lack of real-time clinical data such as survival and recurrence rates. Overall, the retrospective experimental design, imbalance between normal and OSCC sample sizes, and lack of multivariable analysis and external validation have limited the scope of the study. We envision that follow-up clinical studies can closely examine the correlation of HSP27 expression with recurrence and survival rates, which would help to firmly establish HSP27 as a molecular biomarker for oral cancer.

## Conclusions

In this study, we documented a significant upregulation in HSP27 expression in OSCC in comparison to normal oral mucosa. Additionally, HSP27 expression is correlated with the histopathological grade, which is important as higher histopathological grades are associated with more aggressive behavior in tumors. The expression of HSP27 is also correlated with the nodal status, as well as the overall clinical stage, which has a direct association with the five-year survival rate. These findings are particularly significant since they all point to an increased HSP27 expression in patients with a diminished survival outlook as indicated by other clinical parameters, further highlighting the prognostic indicator status of HSP27. Although this study is retrospective by design, it paves the way for the potential application of HSP27 as a supplementary diagnostic and prognostic tool in evaluating and monitoring disease progression in patients suffering from oral cancer and subsequent prospective longitudinal studies looking at larger cohorts of patients along with survival and treatment outcome follow-up data.
